# Is having your cell phone the key to happiness, or does it really matter? Evidence from a randomized double-blind study

**DOI:** 10.1186/s40359-024-01595-y

**Published:** 2024-02-26

**Authors:** Todd McElroy, William Young

**Affiliations:** 1 Psychology Department, Florida Gulf Coast University, The Water School, Ft. Myers, FL USA; 2 Florida Gulf Coast University, Ft. Myers, FL USA

**Keywords:** Affect, Cell phone, Technology

## Abstract

**Background:**

Affect can influence people’s perceptions, decisions, and the way they make sense of an experience. Some studies show that having one’s cell phone removed will lead to negative emotional reactions, while others have found no significant impact on how we feel. In this paper we investigate the impact of cell phone possession and removal on participant’s affective state.

**Methods:**

We use a randomized double-blind procedure to examine whether cell phone removal enhances negativity, promotes positivity, or is emotionally inconsequential. We measure affect using a PANAS self-report scale as well as a less transparent temporal-estimation procedure.

**Results:**

Our findings suggest that cell phone possession or removal has no influence on a person’s affective state.

**Conclusions:**

Measured through both the PANAS self-report scale and temporal estimation task, affect remained consistent regardless of cell phone possession. These results suggest that cell phones may not carry the emotional weight often attributed to them. This finding challenges a common theme revolving around the negative emotional impact of cell phones and technology. Consequently, these findings may have important implications for the generally perceived notion that cell phones are having a negative effect on people’s emotions.

## Introduction

The pervasive use of cell phones has led to an increased dependence on these devices in everyday life. People find themselves constantly distracted by the urge to check their cell phone [1]. Studies reveal that the mere presence of a cell phone can be distracting, regardless of its active use or physical interaction with the user [2]. Given the prevalence of cell phones, researchers are increasingly exploring how both the presence and absence of cell phones influence emotional well-being.

Affect refers to an individual’s emotional or feeling component, encompassing emotional states, from joy and pleasure, to sadness and anger, and ranges from positive to negative [3]. It is regarded as a critical element of human experience, influencing both cognitive and behavioral processes. For example, research has shown that people’s moods can influence their judgments and decisions, this can be seen with people in positive moods making more optimistic decisions than those in a negative mood [4]. Research has also shown that affect can influence the way people perceive information. For instance, one study found that people in a negative mood were more likely to make dispositional attributions to negative events. In contrast, people in a positive mood were more likely to make situational attributions to negative events. This trend, alongside related research, indicates affect’s pervasive role across life’s many facets. It not only shapes our perceptions [5] but also influences our behaviors [6] and social interactions [7], fundamentally altering the way individuals interpret and engage with their experiences.

Measuring emotions or affect poses a considerable challenge [8]. Self-report scales serve as a mirror, reflecting a person’s inner emotional landscape, capturing the nuances and subtleties of their affective state. One way that affect has been shown to influence us is through time perception [9] which has been shown across a wide range of contexts. For example, Studies using basic emotional stimuli like sounds have found that negative valenced sounds tend to make temporal durations seem longer [10]. Temporal duration is overestimated when viewing faces with negative affect, particularly angry and fearful expressions, [11]. People often perceive negatively charged emotional experiences as lasting longer than neutral ones [12], and even the experience of listening to pleasant music seems shorter in duration compared to when one listens to unpleasant music [13]. These studies suggest that affect and arousal influence attentional time sharing leading to differences in our “internal clock’s speed”, which changes our time perception [9]. Thus, there is substantial findings that support the general idea that positive and negative emotions influence time perception, making time feel shorter when we are enjoying ourselves and longer when we are feeling badly.

### Studies investigating cell phone possession and affect

As research shows, emotions significantly influence time perception. This raises an intriguing question about the role of cell phones in shaping our emotional landscape. Does frequent cell phone use extend to negative or positive emotions? Newly emerging research has begun to focus on our interactions with technology and understanding consumer interactions with automated devices, including emotional ones. One critical component is the necessity of considering the psychological aspects in these interactions, thereby contributing to a more comprehensive understanding of the dynamic relationship between consumers and the automated technologies embedded in everyday devices such as cell phones [14]. This literature presents a complex picture of the effects of cell phone use, it is characterized by varied methodologies and focus areas. A comprehensive review of chronic outcomes of cell phone addiction [15] found that an excessive usage pattern adversely impacts personal, social, and professional spheres. This review discusses the prevalence, root causes, symptoms, and associated side effects such as anxiety, depression, and sleep disturbances, highlighting the necessity for further investigation in this area.

In a noteworthy departure from previous research, a recent study [16] offers a fresh perspective on the short-term effects of cell phones. Conducted in a controlled environment, this study is pivotal in exploring how cell phones influence tasks that demand visual attention. Visual attention is a critical aspect of cognitive processing in our increasingly screen-oriented world. One of the most intriguing findings from this study is the suggestion that the presence of a cell phone may not necessarily hinder, but could in fact subtly enhance, performance in specific tasks. This counterintuitive result not only challenges existing assumptions but also adds a novel dimension to our understanding of the interaction between technology and cognitive function. Our current study compliments this recent research by highlighting the question of affective influence.

In a study investigating the immediate effects of cell phone separation [17], researchers investigated the affective response when cell phones were removed from participants for a duration of twelve hours. Contrary to what might be expected, this study found no significant difference in anxiety levels between the group separated from their cell phones and the control group. This finding indicates a potentially minimal affective impact of short-term cell phone separation.

In a closely related study that offers a different perspective [18], researchers examined physiological responses to a cell phone ringing when out of a person’s possession. The study reports increased physiological arousal, anxiety, and unpleasantness when the cell phone rang in a visible location but was not within the participant’s possession. This finding, contrasted with scenarios where the cell phone was in possession but silent, suggests a nuanced relationship between cell phone accessibility and affective states.

Another aspect of cell phone possession is explored in a recent study [19], which connects cell phone use with emotional regulation and personality traits. This study reveals that individuals who struggle with emotional regulation are more likely to use cell phones as coping mechanisms during negative emotional states, potentially leading to negative affective responses when deprived of their cell phones.

Collectively, these studies paint a diverse picture of the impact of cell phone possession, use and separation. The apparent contradictions in their findings can be attributed to differences in what was measured (such as affect or task performance), the context of cell phone use (active use, passive presence, or complete absence), and the duration of the study (short-term laboratory settings versus long-term daily life scenarios). In light of this varied research landscape, our study was designed to help add clarity for how cell phone possession and removal affect an individual’s emotional response.

Our study’s investigation into the relationship between cell phone possession and affect implicitly explores the impact on time perception. Previous research suggests that our perception of time can be influenced by our emotional state. In this context, we reasoned that the removal of a cell phone, a device assumed to be closely tied to our daily emotional experiences, might alter an individual’s affective state, thereby influencing their perception of time.

**Hypothesis:**

Amidst the increasing reliance on cell phones in daily life, a critical question emerges: does the removal of a cell phone from an individual’s possession lead to a significant change in their affective state? This current study explores the affective consequences of cell phone removal, considering the growing dependence on these devices and their potential role in shaping emotional experiences. While existing research offers conflicting views, with some studies indicating that cell phone removal leads to negative affect, and others suggesting minimal impact, this study seeks to provide a clearer understanding of this dynamic.

Our primary objective is to investigate the emotional impact of cell phone possession and removal. To this end, we hypothesize that the removal of a cell phone from a participant will lead to an increase in negative affect and a decrease in positive affect. This hypothesis will be measured using the Positive and Negative Affect Schedule (PANAS) self-report scale, providing a quantifiable assessment of the participant’s emotional state following the removal of their cell phone. As a secondary measure of affect, potentially triggered by cell phone removal, we will examine participant’s temporal estimates during cell-phone removal. By addressing this hypothesis, our study contributes to the broader understanding of how integral personal technology is to our emotional well-being and daily functioning.

## Method

### Participants and design

The sample consisted of 151 participants (87 females), 18–34 years old who were recruited from a Psychology Department Recruitment Pool and received class credit for participation^1^. All participants provided written informed consent prior to participating in the study. The study was approved by the Institutional Review Board at Florida Gulf Coast University (IRB# 2021-57). The study’s hypothesis was preregistered in Open Science Framework (https://osf.io/swxh7/) and the raw data is publicly available (https://osf.io/xfkh8).

The study utilized a between-subjects design with three conditions: Cell phone in possession and on, Cell phone in possession but turned off and put away, and cell phone removed from the room. Participants were randomly assigned to one of the three cell phone conditions. Each condition contained the same set of instructions and stimuli, but the instructions presented by the blinded researcher varied. The study had two dependent variables, the first dependent variable was participants change score for affect, the second dependent variable was participant’s reported time interval estimation.

### Materials

In this study, we utilized two measures to assess affect, the Positive and Negative Affect Schedule (PANAS; [20]) and temporal estimation. The PANAS measure consisted of 20 self-report items measuring positive affect (e.g., interested, excited, alert) and negative affect (e.g., distressed, upset, guilty). Participants were asked to rate the extent to which they experienced each feeling on a 5-point Likert scale ranging from 1 (very slightly or not at all) to 5 (extremely). The PANAS has demonstrated high internal consistency and test-retest reliability [20].

The second measure was a temporal estimation question, which assessed participants’ estimate of the duration of time Researcher Assistant 2 was out of the lab room. The question was presented in the following way: “Precisely how long would you estimate the researcher was just out of the room? Please mark your estimate and try to be exact.” Participants responded on a scale ranging from 0 to 10 min, with higher scores indicating a longer estimate. This type of temporal estimation task has been shown to be a reliable and valid measure of time perception in previous research [21] and the 3 min interval used for time estimation in this study is in line with prior research using similar methodologies to measure temporal estimation differences associated with affect [22, 23].

### Procedure

To adhere to the double-blind protocol, the researchers worked independently throughout the experiment. When participants arrived to the study, they were greeted by the researchers who introduced themselves. In the beginning, Research Assistant 1 (RA1) invited the participant to the lab room where the experiment took place. RA1 made the participant comfortable, giving them an overview of the experiment informing them that it would take place in “this room” and clarifying that if they (the participant) should feel uncomfortable or want to leave at any point during the experiment, then they may stop participating without penalty. The RA1 then provided the participant with informed consent, then informed them that the other Research Assistant 2 (RA2) would be in shortly, RA1 then left the room to go into an adjoining room to prepare for the next part of the experiment, at which time they used a prearranged randomized order to determine which condition the participant would be placed into. At this time RA1 notified RA2 that they are ready to begin their part of the experiment. While RA1 remained in a separate room, RA2 entered the experimental room and introduced themselves to the participant to make them feel more comfortable. RA2 informed the participant that they would first like to get their opinion on a few questions and ask them to perform a few simple tasks. The first item presented to participants was the PANAS (Note: directed toward the “moment” evaluation of affect) measure. After the participant completed this task, RA2 provided them with an unrelated filler task^2^. This filler task was only used as a buffer before the manipulation and after the PANAS administration. The participant is given four minutes to complete these tasks, RA2 then removed the tasks, informed the participant that RA1 will be returning, and left the room. RA2 then notified RA1 that they had completed their part of the study and RA1 again returned to the room. At this time, RA1 moved to the manipulation phase of the experiment and implemented the cell phone condition that was determined from a randomized list to which only they had access. RA1 then presented the participant with one of the three experimental conditions. RA1 entered the room and presented the participant with one of three conditional scripts: “For the remainder of the experiment, please”:

Condition 1: “Keep your cell phone on but we ask that you put it away for the remainder of the experiment” (cell-phone on and in possession).

Condition 2: “Turn your cell phone off and put it away for the remainder of the experiment” (cell phone off and in possession).

Condition 3: “Turn your cell phone off, I need to remove it for the remainder of the session, I will keep it in a safe location and once the study is complete I will return it to you” (cell phone removed from possession).

After completing the cell-phone manipulation, RA1 then left the room. To ensure the integrity of the double-blind study, RA1 consistently brought a bag into and out of the lab room, irrespective of the experimental condition. This consistent action was done to conceal whether a phone was being removed from the room– a key aspect of the third condition. Specifically, the bag’s primary role was to discreetly transport the phone out of the room in the third condition, preventing RA2 from deducing the experimental condition based on the presence or absence of the bag.

After leaving the room, RA1 informed RA2 that they had completed that part of the study and RA2 could then enter the room. RA2 returned to the lab room and provided participants with the second PANAS measure to complete (also directed toward the “moment” evaluation), followed again by a second filler task. After participants had worked for four minutes and completed the second PANAS measure, RA2 informed them that they had completed this part of the session and they (RA2) would return shortly. At this point, RA2 waited until the lab room door closed completely at which point they started a stop-watch timer. After exactly 3 min, RA2 reentered the lab room with the final task for the participant. This task asked the participant how long they thought RA2 was out of the lab room. After the participant filled out the final task, RA2 then left the experimentation room and RA1 reentered the room and debriefed the participants, asked if they have any questions and concluded the experiment.

## Results

The study was preregistered in Open Science Framework (https://osf.io/swxh7/) on 10/26/2021 prior to conducting the study. Separate one-way ANOVAs were conducted to analyze the effects of cell phone possession on affect (PANAS scores) and temporal estimation. We report PANAS change scores (Pre-Post) for both positive and negative affect measurements. This score is determined by the difference between PANAS score 1 (before the cell phone manipulation) and PANAS score 2 (after the cell phone manipulation). When the finalized data were recorded, five participants were not included in the dataset because they did not complete the entire study, and two participants chose not to participate. The data from this study is publicly available (https://osf.io/xfkh8).

### Affect score

The first ANOVA revealed a non-significant effect of cell phone status on positive affect difference scores, *F* (2, 141) = 0.1, *p* =.9, ηp2 = 0.001 (see Table 1). Post-hoc tests using Tukey’s HSD indicated that participants in the cell phone removed condition (M = 1.69, SD = 3.7) reported similar affect change scores to those in the cell phone possession and turned on condition (M = 1.89, SD = 4.7), *p* =.96, and those in the cell phone possessed but turned off condition (M = 1.52, SD = 3.11), *p* =.98. No significant difference was found between the cell phone possession and turned on condition and the cell phone possessed but turned off condition, *p* =.9.

**Table 1:** Means and standard deviations for positive and negative differences and time estimates by condition

		Positive difference	Negative difference	Time estimate
Cell phone status	N			
Possessed and on	44	1.89 (4.70)	0.61 (2.58)	3.07 (1.26)
Possessed and off	46	1.52 (3.11)	-0.15 (3.18)	2.86 (1.33)
Removed	54	1.69 (3.70)	-0.04 (2.47)	2.98 (1.51)

*Note* Standard deviations are presented in parentheses. N represents the sample size for each condition

The second ANOVA tested for differences in the negative affect change scores from PANAS 1 assessment to PANAS 2. This one-way ANOVA revealed a non-significant effect of cell phone status on negative affect difference scores, *F* (2, 141) = 1.02, *p* =.36, ηp2 = 0.01 (see Table 1). We again used Tukey’s HSD for Post-hoc tests which showed that participants in the cell phone removed condition (M = − 0.037, SD = 2.47) reported similar affect change scores to those in the cell phone possession and turned on condition (M = 0.614, SD = 2.58), *p* =.48, and those in the cell phone possessed but turned off condition (M = − 0.15, SD = 3.18), *p* =.98. No significant difference was found between the cell phone possession and turned on condition and the cell phone possessed but turned off condition, *p* =.39.

### Temporal estimation

The third one-way ANOVA also revealed a non-significant effect of cell phone possession on temporal estimation, *F* (2, 141) = 0.27, *p* =.77, ηp2 = 0.004 (see Table 1). Post-hoc tests using Tukey’s HSD indicated that participants in the cell phone possession and turned-on condition (M = 3.07, SD = 1.26) did not differ in their temporal estimates between those in the cell phone removed condition (M = 2.98, SD = 1.5), *p* =.95 or those in the cell phone possessed but turned off condition (M = 2.86, SD = 1.33), *p* =.75, or between the cell phone removed condition and the cell phone possessed but turned off condition, *p* =. 9.

Building on the preceding analyses, a Multivariate Analysis of Variance (MANOVA) was conducted to further test for any effects of cell phone possession status across the three dependent variables: positive affect change scores, negative affect change scores, and temporal estimation. The results of the MANOVA indicated that there was no significant multivariate effect of cell phone status on these dependent variables, *F* (6, 282) = 0.46, *p* =.83. Further, when examining the between-subjects effects, the analysis revealed no significant effects for positive affect change scores, *F* (2, 141) = 0.10, *p* =.905, negative affect change scores, *F* (2, 141) = 1.024, *p* =.362, and temporal estimation, *F* (2, 141) = 0.268, *p* =.766.

## Discussions

Affect refers to an individual’s feeling component of their experience, and it can influence perceptions, decisions, and the overall way someone makes sense of an experience, including the passage of time. Research has shown conflicting evidence on the impact of cell phone possession on affect, with some studies suggesting that having one’s cell phone removed will lead to a negative emotional response, while others have found no significant impact. In this study we predicted that having one’s cell phone removed would lead to relatively more negative affect and that this negativity will lead participants to perceive a time interval to be longer than when they possessed their cell phone.

The results indicate that having one’s cell phone removed had no impact on affect. Specifically, in our measure of affect, the PANAS, individuals who did not possess their phone reported similar levels of positive and negative affect as those who held their phone but turned it off and those who held their phone and kept it on. Similar results were observed in our secondary measure of temporal estimation.

The current findings present an interesting contrast to previous research that has reported significant changes in affective states. One such study [18] found a significant effect on self-reported unpleasantness and pleasantness when participants were separated from their ringing iPhones, as compared to when they possessed them. This study reported increased unpleasantness and decreased pleasantness when the iPhone was not in possession and was actively ringing. This seeming inconsistency can be reconciled by considering the specific conditions under which the affective changes were observed. The key difference being the active ringing of the iPhone during separation, a variable we did not manipulate in our experiment. The ringing condition could be a critical factor in inducing an affective response, potentially creating a sense of urgency or anxiety from missing an important call.

It is also important to point out that if we focus solely on the conditions where the cell phone did *not* ring, their findings of no significant difference between separation and possession align with our results. This consistency in null findings under similar conditions (non-ringing) suggests that the mere act of cell phone separation may not be emotionally impactful. Therefore, our null findings and the null results under non-ringing conditions of prior research [18] agree and highlight that mere separation from the phone does not appear to significantly impact affective states.

There are several limitations that may have influenced the findings of our study and suggests areas for future exploration. Firstly, the use of the PANAS scale for measuring affect presents a potential limitation. While the PANAS is a reliable tool that is commonly used to assess positive and negative affect, it may not capture the full spectrum of emotional responses that can occur when a cell phone is removed. This raises the possibility that more nuanced or complex emotional reactions might have been overlooked. Future research could address this by incorporating more comprehensive affective measurement tools or qualitative methods to capture a wider range of emotional responses.

Additionally, the controlled laboratory setting of our study may not fully replicate the naturalistic contexts in which individuals interact with their cell phones. The environmental and situational factors in everyday life that influence cell phone use and its emotional impact were not represented in the lab setting. Therefore, the generalizability of our findings to real-world scenarios might be limited. In a similar fashion, our study included the use of self-report questionnaires, a common approach in quantitative research. While this method can raise concerns about the validity of responses, as respondents might lean towards socially acceptable answers [24], it remains a widely accepted tool for initial assessments in psychological research, especially when logistical constraints limit the feasibility of clinical interviews. Future studies could explore these dynamics in more naturalistic settings to better understand the affective impact of cell phone possession and removal in daily life.

Furthermore, our study did not account for individual variations in cell phone usage and dependency. Future research should explore how these individual differences influence cell phone possession and removal, as well as the subsequent affective responses. This includes extending this type of research to a more diverse sample. For example, persons who are more attached to their phones may have greater negative affect when their phones are taken away. Furthermore, future research should investigate how different types of cell phone use (e.g., social media, texting, phone calls) may influence emotional responding and time perception. This could provide insights into how specific cell phone uses, especially social media, may impact emotional experiences and time perception.

Building on the findings and limitations of our study, several avenues present themselves for future research in the area of technology use and emotional well-being. Firstly, considering the limitation of the PANAS scale in capturing the full range of emotional responses to cell phone removal, future studies should explore the use of more comprehensive emotional assessment tools. These could include mixed-method approaches combining quantitative scales with qualitative interviews and neuroscience techniques to capture a more nuanced understanding of emotional responses.

Future research should also consider including individual personality traits to examine how individual differences may influence cell-phone possession. For example, one personality trait, openness-to-experience, can influence emotional resonance of people’s choices and influence their decisions [25]. Research has begun to examine how this trait impacts perceptions in a technology-rich environment. For example, one study [26] demonstrated that individuals with greater openness-to-experience were more inclined to perceive virtual reality (VR) as an effective tool in training scenarios. This concept is further supported by a recent study [27], showing that the interplay between design elements of online knowledge communities and traits like openness-to-experience can influence the extent of serendipitous knowledge acquisition. These findings collectively highlight the importance of including individual differences, such as openness-to-experience, in the design of technologically driven situations.

Additionally, the controlled laboratory setting of our study limits the generalizability of the findings to real-world scenarios. Therefore, future research should aim to investigate the affective implications of cell phone use in naturalistic settings. Longitudinal studies tracking individuals’ emotional states in relation to their cell phone interactions over time could provide insights into the long-term effects of technology use on emotional well-being.

Furthermore, integrating psychological theories related to attachment and dependency could offer a deeper theoretical grounding for understanding the emotional bonds people form with their technology. Research could explore parallels between cell phone attachment and traditional attachment theories, providing insights into the psychological underpinnings of our relationships with digital devices.

Finally, in light of the increasing integration of technology into everyday life, it is essential to explore the broader implications of these findings for mental health and well-being. Future studies should examine how habitual cell phone use, especially in contexts of separation or overuse, relates to broader mental health outcomes [28 ] such as anxiety, stress, and overall life satisfaction.

In conclusion, this study provides valuable insights into the relationship between cell phone possession and affective states. Contrary to widespread belief, our findings suggest that the removal of a cell phone does not significantly impact an individual’s emotional state. The findings from both the PANAS self-report scale and the temporal estimation measures consistently indicate that affect remains unchanged, regardless of cell phone possession. This challenges the common notion that cell phones are integral to our emotional well-being. Further, it questions the emotional dependency often attributed to cell phones, suggesting that our affective responses might be more resilient to the presence or absence of these devices than previously thought. This study adds a new perspective to the ongoing discussion about technology’s role in shaping our emotional experiences, highlighting the need for further exploration into how personal devices influence our psychological state.

## Data Availability

The study was preregistered in Open Science Framework (https://osf.io/swxh7/) and the data are publicly available (https://osf.io/xfkh8).

## Footnotes

1 Targeted Cell size was determined by performing a Power analysis (α = 0.05% Power = 0.8), yielding a cell size of *N* = 44. The hypothesized difference in means was obtained from prior related research [18].

2 The filler task used in this study was a Word Find task which contained a 15 × 15 array of letters. The words were affectively neutral and the same task was used consistently across conditions.
